# Exploitation of Cytotoxicity of Some Essential Oils for Translation in Cancer Therapy

**DOI:** 10.1155/2015/397821

**Published:** 2015-02-04

**Authors:** Rossella Russo, Maria Tiziana Corasaniti, Giacinto Bagetta, Luigi Antonio Morrone

**Affiliations:** ^1^Department of Pharmacy, Health and Nutritional Sciences, Section of Preclinical and Translational Pharmacology, UCADH, University of Calabria, 87036 Rende, Cosenza, Italy; ^2^Department of Health Sciences, University “Magna Graecia” of Catanzaro, 88100 Catanzaro, Italy; ^3^University Consortium for Adaptive Disorders and Head Pain, UCADH, University of Calabria, 87036 Rende, Cosenza, Italy

## Abstract

Essential oils are complex mixtures of several components endowed with a wide range of biological activities, including antiseptic, anti-inflammatory, spasmolytic, sedative, analgesic, and anesthetic properties. A growing body of scientific reports has recently focused on the potential of essential oils as anticancer treatment in the attempt to overcome the development of multidrug resistance and important side effects associated with the antitumor drugs currently used. In this review we discuss the literature on the effects of essential oils in  *in vitro* and *in vivo* models of cancer, focusing on the studies performed with the whole phytocomplex rather than single constituents.

## 1. Introduction

Current therapeutic approaches to cancer are often associated with the development of multidrug resistance, important side effects, and high cost, underscoring the unmet need for more efficacious and less toxic interventions. The vegetal kingdom has always represented an attractive source for therapeutics and several examples do exist for natural products being included in current protocols to tackle the limits of chemotherapy. Accordingly, vincristine, vinblastine, colchicine, taxol, paclitaxel, and others are plant-derived anticancer drugs currently used in clinic [1–3].

Among phytochemicals, essential oils have been considered attractive for their wide variety of bioactivities. Anticancer potential of essential oils has been explored and several studies are now available in the literature. A MEDLINE survey on PubMed for “essential oil and cancer” (November 2014) retrieves 686 results with a remarkable surge in publications over the last 15 years (459 out of 686 studies), while a search for “essential oil and cytotoxicity” reports only 270 results, with 234 published in the last 10 years. These numbers suggest that the studies in this field have been initiated rather lately despite the fact that essential oils have been known since ancient times. The reported studies can be divided into* in vivo* and* in vitro* and are related to essential oils from a wide variety of plants or, mainly, their constituents.

## 2. Essential Oils: A Matter of Chemical Complexity

The term “essential oil” was coined in the 16th century by Paracelsus von Hohenhein that named the effective component of a drug “Quinta essential” [4]. Traditionally, essential oils have been used for their biological activities including antiseptic, analgesic, sedative, anti-inflammatory, spasmolytic, and locally anesthetic properties [5]. Furthermore, they are used in aromatherapy for health improvement due to their sedative or stimulant properties [6, 7].

Essential oils (also called volatile or ethereal oils) are aromatic, highly volatile, hydrophobic liquids produced by aromatic plants as secondary metabolites. To date about 3000 essential oils are known, of which about 300 are relevant for pharmaceutical, agronomic, food, cosmetic, and perfume industries. Aromatic plants sources of essential oils mainly grow in temperate and warm areas, like Mediterranean and tropical countries. Often the geographical areas of growing are restricted and therefore the relevance of some essential oils is partially limited to the local traditional pharmacopoeia.

Essential oils can be synthesized by several plant organs (i.e., buds, flowers, leaves, stem, twigs, seeds, fruits, roots, wood, or bark) and stored in secretory cells, cavities, canals, epidermic cells, or glandular trichomes. Each essential oil is a very complex mixture of molecules, which contains between 20 and 70 components with low molecular weight and at different concentrations. Most molecules are present in traces while two to three are often the most representative components, accounting for 20–70% of the whole oil and responsible for determining the biological activities of the essential oil [5].

Based on their chemical structures, the constituents of essential oils are classified as terpene hydrocarbons, distinct in monoterpenes (C10), sesquiterpenes (C15), and diterpenes (C20); terpenes containing oxygen (terpenoids), such as alcohols, ketones, aldehydes, esters, lactones, and coumarins; and phenylpropanoids and aromatic compounds derived from phenylpropane that occur less frequently. Monoterpenes are the most abundant constituents and, often, the antitumor activity of essential oils has been related to their presence in the phytocomplex [8].

There is an overall high variation in the chemical profile of essential oils depending on the extraction methods, organ used, age and vegetative stage of the plant, the time of the harvest, and the soil composition [9, 10].

What is reported above stresses two important concepts to bear in mind when studying or reporting on the biological effects of essential oils: (1) characterization of their chemical composition, together with a good quality of the phytocomplexes (as reported in analytical monographs of the European pharmacopeia), is fundamental for their appropriate use; (2) the chemical complexity of the phytocomplex contributes to its biological effects, since each constituent takes part in the overall outcome and may modulate the effects of the others.

This often translates into the concept that, in most cases, it is not possible to ascribe the effects of an essential oil to a single component. Therefore, studies on individual ingredients might report results that do not recapitulate the effect of treatment with the phytocomplex as a whole. In view of the latter, in the present paper, we discuss the cytotoxicity and potential anticancer activities of whole essential oils rather than their single constituents.

## 3. Cytotoxicity of Essential Oils: Underlying Mechanisms

As normal cells evolve into a neoplastic state, they acquire new biological activities that are hallmarks of cancer cells. These include sustaining proliferation signaling, evading growth suppressors, resisting cell death, inducing angiogenesis, and activating invasion and metastasis [11]. Therefore, chemotherapy often relies on the characteristics of drugs to reduce the ability of cancer cells to grow and divide and to induce cell damage and death.

With the aim of testing their possible use as alternative or complementary cancer treatments, several essential oils are under investigation for their cytotoxic and antiproliferative actions in cancer cell lines or tumor bearing animals [6, 12]. Different mechanisms may account for the reported cytotoxic effects of essential oils or their constituents. These include induction of cell death by apoptosis and/or necrosis, cell cycle arrest, and loss of key organelles function. Some of these effects are ascribable to the lipophilic nature and low molecular weights of the constituents of essential oils that allow them to cross cell membranes altering the phospholipid layers, increasing membrane fluidity, and leading to leakage of ions and of cytoplasmic content. Reduced ATP production, alteration of pH gradient, and loss of mitochondrial potential are just few of the consequences of disturbed cellular membranes. Furthermore, essential oils can also act as pro- or antioxidants, affecting the cellular redox state [13–15].

## 4. Antitumoral Activity of Selected Essential Oils

### 4.1. *Melissa officinalis L*. Essential Oil

*Melissa officinalis L*. (lemon balm) is a medicinal plant widely diffused in Europe and Mediterranean region. Aqueous and alcoholic extracts are traditionally used for their digestive and antispasmodic [16], sedative [17], antiviral [18, 19], and antioxidant properties [20].* M. officinalis* essential oil has been shown to possess antibacterial, antifungal, and spasmolytic activities [21–23], while antitumoral effect has only recently been reported.

In 2004, de Sousa and colleagues investigated [20], by MTT assay, the cytotoxic activity of* M. officinalis* essential oil in lung (A549), colon (Caco-2), breast (MCF-7), and leukemia (HL-60 and K562) human cancer cell lines and in a mouse melanoma cell line (B16F10). In this study, dilutions of the essential oil ranging from 1 : 50.000 to 1 : 2.000 induced a dose-dependent inhibition of cell viability in all tested tumor cell lines, although each culture showed different sensitivity [20]. The antitumoral effect of* M. officinalis* was later studied in human glioblastoma multiforme cell lines; in U87 and A172 cultures, treatment with the essential oil for 48 h decreased cell number in a dose-dependent manner and this was associated with induction of apoptosis, as demonstrated by the presence of DNA fragmentation and activation of caspase-9 and caspase-3 [24]. Glioblastoma multiforme (GBM) is the most common and aggressive form of glioma, and tumor cell drug resistance limits a successful treatment worsening the prognosis. Expression of members of the multidrug resistance related proteins (MRP), which belong to the ATP binding cassette (ABC) transporter superfamily, is one of the mechanisms contributing to the GBM chemoresistance. Interestingly, treatment with the monoterpene citral, that represents more than 85% of the* M. officinalis* essential oil and reproduces the cytotoxic features of the essential oil, reduces the activity and downregulates the expression of MRP1 in GBM cell cultures that express an active form of the transporter [24].

The ability of* M. officinalis* essential oil and of citral to induce apoptosis in resistant cells that express MRP1 suggests their potential for tumor treatment.

### 4.2. *Melaleuca alternifolia* (Tea Tree) Oil

Tea tree oil (TTO) is the essential oil steam distilled from* Melaleuca alternifolia* of the Myrtaceae family, a plant native from Australia. Traditionally, the oil was used by aboriginal Australian for insect bites and skin infections and rediscovered in 1920s for its topical antiseptic effects. To date, the essential oil is part of several products for skin and wound care and its safety/toxicity associated with topical uses has been rigorously examined [25]. TTO consists largely of monoterpenes; about half are oxygenated and the rest are hydrocarbons [26]. Out of the over 100 components of the oil, the more represented are terpinen-4-ol, *γ*-terpinene, *α*-terpinene, 1,8-cineole, and *ρ*-cymene. Currently, the composition of TTO must adhere to an international standard for “Oil of* Melaleuca* terpinen-4-ol type” which sets the upper and lower limits for 14 components of the oil although it does not indicate the species of* Melaleuca* that must be the source of the oil (International Organization for Standardization, ISO 4730: 1996) [25]. Terpinen-4-ol, the most abundant component of the oil, is thought to be the main active constituent responsible for the several* in vitro* and* in vivo* activities reported for TTO [27]. TTO is known for its antibacterial [28, 29], antifungal [30], antiviral [31], and anti-inflammatory properties [32], while only recently the phytocomplex and some of its components have been screened for anticancer activities [33–35].

Studies evaluating cytotoxicity of TTO on cultured cells were initially performed to determine its potential toxic effects. TTO toxicity was tested on a wide panel of human cell cultures including cervical cancer (HeLa), acute lymphoblastic leukemia (MOLT-4), erythromyeloblastoid leukemia (K562), B cell derived from bone marrow of a patient with acute myeloid leukaemia (CTVR-1), fibroblast, and epithelial cells. In these studies TTO showed an IC50 on cell growth ranging from 20 to 2700 *μ*g/mL [31, 34, 36, 37].

The potential antitumoral activity of TTO was reported in a study by Calcabrini and colleagues (2004) in human melanoma M14 wild type cells and their drug-resistant counterparts, M14 adriamycin-resistant (ADR) cells. TTO, at the higher used concentrations (0.02 and 0.03%), as well as terpinen-4-ol, was able to inhibit the growth and induce caspase-dependent apoptotic cell death in both wild type and drug-resistant melanoma cells with the latter being more susceptible to the cytotoxic effect [35]. The authors suggested that the greater sensitivity to the TTO treatment displayed by the drug-resistant cells could be ascribed to the different lipid composition of the plasma membrane since there is evidence indicating that multidrug resistance phenotype is also associated with changes in membrane lipid composition [38, 39]. This would suggest that, as claimed for the antimicrobial effect, the cytotoxicity of TTO might be due to the interaction of the lipophilic components of the oil with the phospholipid bilayer of cell membranes with consequent alteration of cell growth and activity. It is worth noting that an earlier study testing the cytotoxic effect of TTO on “normal” epithelial and fibroblast cells, having similar susceptibilities as basal keratinocytes to topical agents [40], did not report toxic effects at concentrations that were shown to affect melanoma cell survival [36], thus confirming a higher sensitivity of tumor cells as compared to normal cells.

Cytotoxic effect of TTO has been reported in murine mesothelioma (AE17) and melanoma (B16) cell lines though slightly different IC50 was reported, probably due to the different cell types [41]. In this case, TTO and terpinen-4-ol induced time-dependent cancer cell cycle arrest and cell death by primary necrosis and low levels of apoptosis; differential dose-response between tumor and nontumor fibroblast cells was shown suggesting that TTO might elicit its effect by inhibiting rapidly dividing cells more readily than slower growing noncancerous cells [41]. More recently, the ability of TTO and its major component, terpinen-4-ol, has been also reported to interfere with the migration and invasion processes of drug-sensitive and drug-resistant melanoma cells [33].

Two recent studies investigated the efficacy of topical TTO on aggressive, subcutaneous, chemoresistant tumors in fully immune-competent mice [42, 43]. The studies showed that topical treatment with 10% TTO, given once a day for 4 consecutive days, induced a significant, though temporary, regression of established subcutaneous AE17 tumors and slowed the growth of B16-F10 tumors. Use of DMSO as a penetration enhancer, at concentrations devoid of toxic effects, was necessary to induce the antitumor effect; no effects were evident when using neat TTO or solvents other than DMSO (i.e., isopropanol or acetone). Similar effects on tumor growth were obtained using a combination of the five major components of TTO (terpinen-4-ol, *γ*-terpinene, *α*-terpinene, 1,8-cineole, and *ρ*-cymene) at equivalent doses to those found in 10% TTO but not with the single components [41]. The antitumor effect of topical TTO was accompanied by skin irritation that, unlike other topical chemotherapeutic agents, resolved quickly and completely.

A follow-up study investigated the mechanism of action underlying the antitumor activity of topical TTO reporting that topically applied 10% TTO induced a direct cytotoxicity on subcutaneous AE17 tumor cells, which was associated with nontumor specific activation of local immune response (i.e., neutrophils, dendritic, and T cells) [43]. In particular transmission electron microscopy analysis of AE17 tumor sections from mice treated topically with TTO revealed loss of cellular organization with increased intercellular spaces and accumulation of cell debris, nuclear shrinkage, chromatin condensation, mitochondria swelling and loss of cristae and membranes, significant alteration of endoplasmic reticulum, and less defined cellular membranes. These findings would suggest that, following* in vivo* TTO treatment, tumor cells undergo primary necrosis as previously suggested* in vitro* [35, 42]. Interestingly the topical treatment does not seem to affect the fibroblast adjacent to damaged tumor cells, nor lymphocytes within tumor sections and skeletal muscle fibers adjacent to tumor suggesting that normal cells might have higher tolerance to TTO cytotoxicity as compared to tumor cells.

### 4.3. Essential Oils of Sage

*Salvia* (commonly called sage) is probably the largest genus among the Lamiaceae family, consisting of about 900 species widely distributed throughout the world. The plant mainly grows in temperate, subtropical, and tropical region with Mediterranean, Central Asia, Mexico, Central and South America, and Southern Africa being the major centers.

As suggested by its Latin name, meaning “to save or to cure,”* Salvia*, and in particular the species* S. officinalis*, has been known and utilized for hundreds of years in traditional medicine to treat fever, rheumatisms, perspiration, sexual debility, infection and inflammation of throat and mouth, chronic bronchitis, and mental diseases [44]. Furthermore, sage leaves and its essential oil have carminative, antiseptic, antispasmodic, astringent, and antidiaphoretic properties [45].

The content profile of salvia essential oil defined by the standard ISO 9909 (ISO, 9909, Oil of Dalmatian Sage (*Salvia Officinalis L.*), American National Standards Institute (ANSI), New York, NY, USA, 1997) is the following: *α*-thujone (18–43%), *β*-thujone (3–8.5%), camphor (4.5–24.5%), 1,8-cineole (5.5–13%), humulene (0–12%), *α*-pinene (1–6.5%), camphene (1.5–7%), limonene (0.5–3%), linalool (free and esterified (1% maximum)), and bornyl acetate (2.5% maximum).

However, the essential oil does not always match the standard profile. This is due to the several species-specific differences as well as the influence of genetic and environmental factors, climate conditions, time of sample harvesting, and culture site [46].

Cytotoxic and antiproliferative activities have been reported for essential oils from the aerial parts of several* Salvia* species.* S. officinalis* essential oil exerted cytotoxic activity on C32 amelanotic melanoma and ACHN renal adenocarcinoma human cell lines, with an IC50 of 367 and 108 *μ*g/mL, respectively. Conversely, no effects were reported when the essential oil was tested on MCF-7 human breast cancer and LNCaP hormone dependent prostate carcinoma cell lines [47]. The study did not find significant correlation between the activity of the essential oil and its, commercially available, main component 1,8-cineole, underlying the concept that different components might act together to enhance the observed effect. Accordingly, a study on* S. libanotica*, a different species of sage, reported that three bioactive components of the oil, that is, linalyl acetate, *α*-terpineol, and camphor, synergize inducing cell cycle arrest and apoptosis in human colon cancer HCT-116 p53^+/+^ and p53^−/−^ cell lines [48]. However, the 50% growth inhibition (IC50) observed upon combining the three components (10^−3 ^M) was lower as compared to the effect of the whole oil in SP-1 mouse papilloma cell lines (50 *μ*g/mL) [49], L929sA mouse fibrosarcoma cells (180 *μ*g/mL), and MDA-MB 231 metastatic human breast carcinoma cells (290 *μ*g/mL) [50]. In this case a differential sensitivity of various cancer cell lines to the essential oil must also be taken into account. Interestingly, the study by Itani and colleagues showed that cancer cells have higher sensitivity to the oil, since the growth suppression following exposure to the combined components had only minimal effects on normal human intestinal cells [48].

Cell viability of squamous human carcinoma cell line of the oral cavity (UMSCC1) was significantly reduced following treatment with* S. officinalis* essential oil and this was associated with changes in the expression of genes involved in aryl hydrocarbon receptor signaling, cell cycle regulation, and p53 signaling [51].

Cytotoxicity of* S. bracteata* and* S. rubifolia* essential oils was reported in M14 human melanoma cells; apoptotic induction in tumor cells was observed at concentrations nontoxic in normal cells [52]. Similar results were obtained in A375, M14, and A2058 human melanoma cells exposed for 72 h to eighteen* S. officinalis* essential oils obtained from different sites in south-central Italy [53]. A different species, common in traditional Chinese medicine,* S. miltiorrhiza*, was shown to have cytotoxic effects in a human hepatoma cell line, inducing depletion of glutathione, reduction of mitochondrial potential, and, in turn, apoptotic cell death [54].

### 4.4. Thyme Essential Oil

Thyme belongs to the Lamiaceae family; due to its wide spectrum of pharmacological properties, it has been used in traditional medicine for thousands of years in countries of the Mediterranean basin. Essential oil of the most studied species,* Thyme vulgaris*, and its principal component thymol has been shown to have antifungal, antibacterial [55, 56], and antioxidant [57] activities. Therefore, thyme is usually employed as expectorant in upper respiratory tract infection, and thymol is often the main antiseptic ingredient in mouth rinses against gingivitis.

In 2007, Ait M'Barek and colleagues tested the cytotoxic effect of Moroccan endemic thyme (*Thymus broussonetii*) essential oil in human ovarian adenocarcinoma cell line (IGR-OV1) and its parental cell line resistant to three chemotherapeutic drugs currently used to treat the ovarian adenocarcinoma (adriamycin, vincristine, and cisplatinum) [58]. In this study all cell lines were sensitive to the cytotoxic effects of the essential oil, although they had a different degree of sensitivity reporting an IC50 ranging between 0.39 and 0.94%; importantly, the authors also showed that administration of the essential oil at the tumor site for 30 days in tumor bearing DBA-2 (H_2_^d^) mice inhibited tumor proliferation, reduced tumor volume, and delayed mouse mortality [58].

In human UMSCC1 head and neck squamous cell carcinoma (HNSCC) cells subtoxic concentrations of* Thymus vulgaris* essential oil stimulated proliferation and viability, while, at higher concentrations, dose-dependent cytotoxic effects were found [59]; under this experimental setting, the observed cytotoxicity induced by the essential oil was associated, as shown by a microarray-based mRNA expression profiling and pathway analysis, with the regulation of three pathways, namely, the interferon signaling, N-glycan biosynthesis, and ERK5 signaling that could be all involved in the effect of thyme essential oils on cancer cell growth and survival [59]. Interestingly, a recent study testing the cytotoxicity of ten essential oils (mint, ginger, lemon, grapefruit, jasmine, lavender, chamomile, thyme, rose, and cinnamon) identified thyme as the most effective on human prostate carcinoma (PC3), human lung carcinoma (A549), and human breast cancer (MCF7) cell lines [60].

### 4.5. Bergamot Essential Oil

Bergamot essential oil (BEO) is a well-known plant extract, obtained by cold pressing of the epicarp and, partly, of the mesocarp of the fresh fruit of bergamot (*Citrus bergamia*, Risso et Poiteau). The fruit belongs to the genus* Citrus* of the Rutaceae family and grows, almost exclusively, in a restricted area along the coast of Southern Italy.

BEO comprises a volatile fraction (93–96% of total) containing monoterpene and sesquiterpene hydrocarbons and oxygenated derivatives and a nonvolatile fraction (4–7% of total) characterized by coumarins and furocoumarins [61, 62]. The most abundant components of the essential oil are the monoterpene hydrocarbon d-limonene and the monoterpene ester, linalyl acetate, with d-limonene accounting for about 40% of the whole oil [63, 64].

Bergamot essential oil has been used by folk medicine as antiseptic and antihelminthic and to facilitate wound healing and these uses are now supported by experimental data reporting the antifungal [65] and antimicrobial [66] activities of the phytocomplex as well as its ability to increase oxidative metabolism in human polymorphonuclear leukocytes [67]. Recently, analgesic [68–70], anxiolytic [71], and neuroprotective [72, 73] effects have been ascribed to bergamot essential oil and these are consistent with the use of the oil in aromatherapy for the relief of pain and symptoms associated with stress-induced anxiety and depression. Furthermore it has been shown in rodents that BEO affects synaptic transmission by modulating the release of specific amino acid neurotransmitters [74] and it produces a dose-related sequence of sedative and stimulatory behavioral effects in freely moving, normal rats [75].

Despite the number of studies on the effects of bergamot essential oil under pathological or normal conditions, data regarding its potential activity on tumor cells have only recently been gained. Accordingly, a recent study reported that exposure of human SH-SY5Y neuroblastoma cells to 0.02 and 0.03% bergamot essential oils significantly reduced cell viability inducing both necrotic and apoptotic cell death; cytotoxicity induced by the phytocomplex was accompanied by cytoskeletal alteration, mitochondrial dysfunction, caspase-3 activation, DNA fragmentation, plasma membrane damage, and cleavage of prosurvival proteins [76]. The mixed features of necrotic and apoptotic cell death induced by bergamot essential oil might be related to its complex phytochemical composition, suggesting that different components might activate different pathways to execute cell death. A follow-up study engaged to identify the components responsible for cell death induced by the phytocomplex showed that, at comparable concentrations with those found in cytotoxic concentrations of the oil, none of the tested constituents (d-limonene, linalyl acetate, linalool, *γ*-terpinene, *β*-pinene, and bergapten) reduced SH-SY5Y cell viability, while only the combination of limonene and linalyl acetate was able to induce cell death [77]. Accordingly, the bergapten-free fraction of bergamot essential oil was shown to be more effective than the complete phytocomplex suggesting that bergapten is not the main component responsible for the observed cytotoxicity [78].

Interestingly, it was recently shown that bergamot essential oil and its main component limonene activate autophagy in SH-SY5Y human neuroblastoma and MCF7 human breast cancer cell lines [79]. This effect was concentration-dependent, unrelated to the effects elicited by the essential oil on cell survival, and occurred with a mTOR-independent mechanism [79]. In view of the role of autophagy in limiting cancer development while facilitating advanced tumor progression [80], the finding that an essential oil is able to activate this pathway can be extremely relevant for its potential application as chemotherapeutic and therefore it stimulates further studies.

As for other essential oils, the hydrophobic nature of bergamot essential oil requires the use of solvents endowed with toxic effects (i.e., DMSO, ethanol) that can limit the therapeutic use of the phytocomplex. Celia and colleagues (2013) recently showed that this limitation could be overcome by loading the essential oil in pegylated liposomes; in addition, the liposomal formulation of bergamot essential oil showed enhanced cytotoxic effect in neuroblastoma cells as compared to the free phytocomplex [78]. Similarly, encapsulation of other essential oils in nanocarriers (i.e., polymeric nanoparticulate formations and lipid carriers, such as liposomes) might represent a good strategy for improving water solubility and stability of essential oils while lowering their effective dose and limiting potential side effects [81].

### 4.6. Other Essential Oils

In addition to the essential oils discussed above, several others have shown anticancer activity* in vitro* and* in vivo*.

The essential oil of* Artemisia annua* L. (commonly known as sweet wormwood) induced apoptosis in SMMC-7721 hepatocarcinoma cells [82] and a number of human cancer cell lines, including melanoma and breast and ovarian cancer, showed hallmarks of apoptosis when treated with the essential oil of the conifer tree* Tetraclinis articulata* [83].

The essential oil of* Cymbopogon flexuosus* (Eastern lemon grass) showed dose-dependent cytotoxicity in colon (502713), neuroblastoma (IMR-32), liver (Hep-g-2), and cervix (SiHa) cell lines with an IC50 value ranging from 4.2 to 6.5 *μ*g/mL. The same oil induced dose-dependent growth inhibition, decrease in the ascitic fluid volume, and total ascites cell count in solid and ascitic Ehrlich and S-180 tumor models in mice [84].

A study conducted by Loizzo and colleagues (2007) compared the cytotoxic activity of five essential oils from Labiatae and Lauraceae families (*Sideritis perfoliata*,* Satureja thymbra*,* Salvia officinalis*,* Laurus nobilis*,* and Pistacia palaestina*) on a panel of cancer cell lines; all the tested oils inhibited tumor cell growth, with* Laurus nobilis* exerting the highest activity on C32 amelanotic melanoma and ACHN renal cell adenocarcinoma [47].

Maggi and colleagues reported the cytotoxic effect of the essential oil extracted from the leaves of* Vepris macrophylla*, a high evergreen tree endemic of Madagascar [85]. The results showed that the essential oil exhibited strong inhibitory effects on MDA-MB 231 human breast adenocarcinoma and HCT116 human colon carcinoma tumor cell lines, with inhibition values comparable to those of the anticancer drug cisplatin [85].

*In vitro* and* in vivo* anticancer effects of the leaf essential oil of* Xylopia frutescens*, a medicinal plant found in Central and South America, Africa, and Asia, were recently reported [86]. MTT assay was used to assess essential oil cytotoxicity in ovarian adenocarcinoma (OVCAR-8), bronchoalveolar lung carcinoma (NCI-H358M), and metastatic prostate carcinoma (PC-3M) human tumor cell lines. Interestingly, in the same study, a seven-day intraperitoneal treatment with the tested essential oil dose-dependently inhibited tumor growth in mice subcutaneously transplanted with Sarcoma 180 cells without reporting evident signs of toxicity [86].

Essential oil of* Nigella sativa* L. (Ranunculaceae family) seeds, known as black seed or black cumin, and its ethyl acetate fractions possess strong, although differential, cytotoxic effects against tumor cells, while butanol extract has limited effects; the reported differential effects for these extracts seem to be related not only to their chemical composition but also to the nature of the tumor cell line tested. Interestingly, minimal cytotoxicity was observed for all the extracts toward normal human peripheral blood mononuclear cells [87]. Furthermore,* Nigella sativa *L. essential oil, when injected at the tumor site of solid tumor bearing mice, significantly inhibited the tumor development, reduced the incidence of liver metastasis, and improved mouse survival [87]. Consistently, previous findings have shown that* Nigella sativa* methanol extract inhibits the growth of Ehrlich ascites carcinoma in mice [88].

## 5. Conclusions

Essential oils have been used in traditional medicine since ancient time; however, research in this area is still in its growing state and a systematic and rigorous approach to the study of biological activities of potential phytotherapeutics is an achievement of the last few decades. This is particularly true for the cytotoxic effects of phytocomplexes. From the available literature essential oils seem to have a great potential as anticancer therapeutic agents; however, information regarding their mechanism of action is still lacking and far from being deciphered.

Indeed, their complex chemical composition makes it difficult to envisage a single mechanism underlying the entireness of the biological effect, which is likely the sum and/or synergy of the biological activity of each component. For the same reason, data obtained from single components may not necessarily be, in turn, applied to the whole essential oil.

On the other hand, the presence in the phytocomplex of numerous constituents that simultaneously interfere with multiple signaling pathway might be the key for overcoming the current limit of chemotherapeutic agents and in particular the development of multidrug resistance.

Although, from the data reviewed in this paper, the use of essential oils in cancer therapy is very promising, the data obtained from* in vitro* and* in vivo* preclinical models have, beside obvious strengths, several limitations and cannot always be fully applicable to humans. Furthermore, administration route, as well as potential toxicity, and side effects are rarely taken into account by the reviewed studies making it difficult to predict the translational potential of those data for the clinical setting.

Therefore, it appears to be of great importance that agencies for research funding (1) continue to support worldwide basic research in the field and (2) stimulate clinical trials for those phytocomplexes where a reasonable wealth of preclinical data is available to limit attrition in late phases of clinical trials. The available European legislation in the field of herbal medicines (Directive 2004/24/EC; [89]) and of clinical trials (regulation number 536/2014) together with the NIH guidelines for definition of anticancer activity of any natural or synthetic substance is the guidance for effective research and development of essential oils in cancer therapy.
